# Challenging presumed technological superiority when working with (artificial) colleagues

**DOI:** 10.1038/s41598-022-07808-x

**Published:** 2022-03-08

**Authors:** Tobias Rieger, Eileen Roesler, Dietrich Manzey

**Affiliations:** Department of Psychology and Ergonomics, Technische Universität Berlin, Marchstr. 12, F7, 10587 Berlin, Germany

**Keywords:** Psychology, Human behaviour, Social behaviour

## Abstract

Technological advancements are ubiquitously supporting or even replacing humans in all areas of life, bringing the potential for human-technology symbiosis but also novel challenges. To address these challenges, we conducted three experiments in different task contexts ranging from loan assignment over X-Ray evaluation to process industry. Specifically, we investigated the impact of support agent (artificial intelligence, decision support system, or human) and failure experience (one vs. none) on trust-related aspects of human-agent interaction. This included not only the subjective evaluation of the respective agent in terms of trust, reliability, and responsibility, when working together, but also a change in perspective to the willingness to be assessed oneself by the agent. In contrast to a presumed technological superiority, we show a general advantage with regard to trust and responsibility of human support over both technical support systems (i.e., artificial intelligence and decision support system), regardless of task context from the collaborative perspective. This effect reversed to a preference for technical systems when switching the perspective to being assessed. These findings illustrate an *imperfect automation schema* from the perspective of the advice-taker and demonstrate the importance of perspective when working with or being assessed by machine intelligence.

Automated systems are rapidly making inroads into our working life and everyday world. From the assessments of private creditworthiness^1,2^ to the evaluation of medical cases^3–5^, automation continuously replaces or at least supports human professionals in their judgments. These automated systems replace stages (i.e., information acquisition and analysis, decision-making, and, in some cases, even action implementation) of human information processing^6^, and, as a result, the role of the human changes from active and solely responsible operator to being less involved in the complete process^7^.

One of the major determinants of successful interaction for both human and technological support is adequate trust towards the respective agent^8,9^. Most crucially, humans have higher performance expectations towards technological aids compared to human counterparts leading to higher initial trust^10,11^. However, if a system failure is experienced, performance expectations are not met for the technical system and both trust and perceived utility are reduced to a level even lower than that for human support. This effect has been coined as “perfect automation schema” and likely occurs because people are aware of their own fallibility but expect machines to work perfectly^10,11^.

Having in mind the possible alarming consequences of the perfect automation schema, it seems negligent to not broaden this view to novel technological developments like artificial intelligence (AI). This is important as even though classical decision support systems (DSS) and AI share many commonalities (e.g., the changed human role^7^ or the lack of systems transparency^1,12,13^), there might also be some key differences in the perception of those systems. First, due to its ability to continuously learn and improve, AI might be perceived as more of an expert system compared to DSS^14^. Thus, even though both kinds of support systems can provide highly reliable recommendations, an AI is likely perceived more competent than a DSS. Second, AI also extends DSS with a higher level of agency and autonomy^15,16^. This might result in the perception that the AI is the deliberate initiator of the actions and their effects. In summary, both continuous learning as well as agency/autonomy might contribute to a higher perceived expertise of AI compared to DSS. Against the background of AI being superior to classical DSS, one would expect this schema to be even more pronounced for AI than for DSS—both with a higher performance expectation and greater trust dissolution in case of errors. Specifically, the presumed expertise and utility^10,11,17^ of an aid is what drives the perfect automation schema, and with a superior system (AI), this should be more pronounced.

Due to AI’s rapid introduction into a plethora of workplaces, new challenges arise and require to go beyond the typically studied outcomes like perceived reliability, trust, and dependence^8,9,18^. The first key aspect emerges from the increased autonomy and agency of AI which poses a new question of perceived responsibility. Even though AI is far from legal responsibility^19^, the highly autonomous and often non-transparent decision-making processes might lead to a change in perceived responsibility^20^. In addition, novel technologies being applied in various contexts also require us to gain a better understanding of people being subject to decisions of technical systems^21^. For example, it seems clear that being the banker who receives automated advice is a different situation from being a loan applicant who asks for a credit. This change of perspective is the second key aspect which the present research tries to address.

The above-mentioned example already illustrates that AI is supporting or replacing humans in tasks which are not limited to quantifiable tasks with a demonstrably correct answer but also in more uncertain tasks where the true state of the world is more ambiguous^22,23^. In summary, we try to close two gaps in prior research. On the one hand, we aim to gain a better understanding of a change of perspective (i.e., from advice-taker to being assessed) that this new technology necessarily entails. On the other hand, we wanted to test and extend possible implications of a perfect automation schema to AI. To do this, we address the role of (artificial) support agents and how they are perceived in various application areas, involving different sorts of tasks. That is, if considering in which variety of areas AI is making its way into, it becomes indispensable to broaden the task view from classical visual detection tasks. For this reason, we started our set of experiments by using a novel real-life application, in which AI has been recently introduced^1,2^. Once it became clear that the perfect automation schema did not hold true in this application, we extended our research using a task similar to the one where a perfect automation schema had been found previously. We continued to systematically vary the task context to both replicate our findings as well as get an understanding of task factors. Specifically, in Experiment 1, we used a complex categorization with personal information (i.e., loan assignment), in Experiment 2, we used a classical detection task with the same personal information as in Experiment 1 (i.e., X-Ray assessment), and in Experiment 3, we used a classical detection task without personal information (i.e., simulated chemical plant). This allowed us to extend our findings and get an understanding of contributing task factors of trust in human-agent interaction.

To ensure good comparability between the experiments, the general structure was the same. That is, it was always the participant’s task to make the final decision after seeing the agent’s recommendation. Moreover, to study the predictions from the perfect automation schema (i.e., more forgiving towards human mistakes), half of the participants experienced an obviously erroneous recommendation from their support agent whereas the other half of the participants received correct recommendations only. After this interaction with the respective agent, participants were asked to rate the following outcomes from the perspective of the advice-taker: perceived reliability, their trust in the support, whether the support took responsibility for its actions, and whether the respective kind of support were useful in this context. Moreover, they were also asked to change the perspective and to rate whether they themselves would like to be judged by the respective support agent in case of the respective context.

Altogether, these experiments were used to extend the knowledge about a classical phenomenon of human-automation interaction by investigating it in the context of novel technologies (i.e., AI), real-life application domains (i.e., loan application, X-Ray assignment, and chemical plant), and with a switch in perspective.

## Results

**Figure 1:**
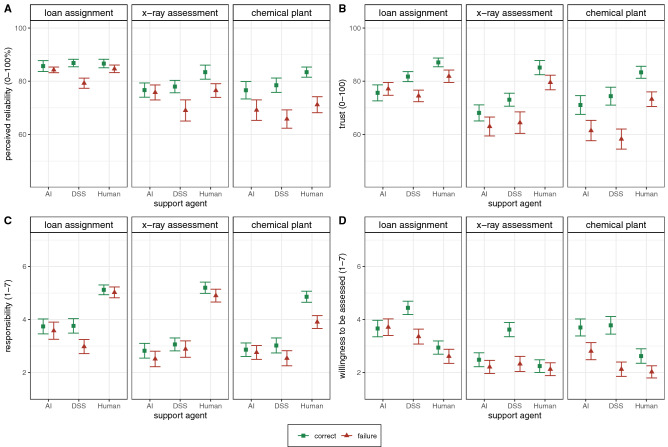
Means and standard errors for (**A**) perceived reliability, (**B**) trust, (**C**) responsibility, and (**D**) the willingness to be assessed by the respective agent as a function of support agent (i.e., AI, DSS, human) and failure condition (i.e., failure vs. all-correct), separately for three experiments with different task contexts (Experiment 1: loan assignment, Experiment 2: X-Ray assessment, Experiment 3: chemical plant). AI = artificial intelligence, DSS = decision support system.

Figure 1 visually illustrates perceived reliability (A), trust (B), responsibility (C) and willingness to be assessed by the respective agent (D), separately for the three different contexts. Results for all other subjective outcomes can be obtained via the open science framework (OSF). For the analysis of all relevant outcomes between-subjects ANOVAs were conducted to investigate main and interaction effects. Moreover, post-hoc tests with a Bonferroni-corrected p-value were used to investigate significant interaction effects or main effects involving a three-level factor (for details see *methods/statistics*).

### Experiment 1: loan decision

As expected, participants who experienced a system failure deviated stronger from the agent’s recommendation ($M=3905.34$) than those who only received correct recommendations ($M=2601.33$), $F(1,275)$ = 6.799, $p$ $= 0.010$, $\eta ^2_G=0.013$. In line with this, the respective support was perceived as more reliable in the correct ($M=86.39$) than in the failure ($M=82.67$) condition, $F(1,275)$ = 7.552, $p$ $= 0.006$, $\eta ^2_G=0.027$. These ratings around 85% are a significant ($t(280)=15.30, p<0.001$) gap between the true experienced and perceived reliability, a common issue with highly reliable systems that can lead to an under-utilization of the support^8^. Surprisingly, the type of support did not play a role for neither the deviation from the system nor the perceived reliability. As reliability is closely linked to subjective trust towards any agent^9^, the fact that trust was descriptively lower after failure experience ($M=77.71$) than when receiving only correct recommendations ($M=81.48$, $p$ $=0.053$) was as anticipated. Remarkably, the assumptions of the perfect automation schema were not met by our data. Instead, we found quite the opposite with higher trust towards human support ($M=84.73$) compared to both the AI ($M=76.35$, $p$ $=0.002$) and the DSS ($M=78.31$, $p$ $= 0.019$), $F(2,275)$ = 6.778, $p$ $= 0.001$, $\eta ^2_G=0.047$. This higher trust towards humans was also not affected by failure experience. Thus, the assumption that people are more lenient towards humans than automation^11,24^ if a failure occurs was not confirmed. This finding contradicts many earlier studies^10,11,24–26^ where technical systems (i.e., automated support agents) were subject of higher performance expectations and trust. In contrast to human interaction partners in earlier studies, our human support was characterized as a subject matter expert. Human support by an expert is of course much more realistic than support by a novice. Note that it is impossible to know to which degree participants believed the experimental manipulation—a limitation which is shared with earlier research^10,11,24–26^.

We assumed that users attribute more responsibility to an AI due to its autonomous learning processes. However, this was not the case. Specifically, more responsibility was attributed to the human ($M=5.08$) compared to the AI ($M=3.66$, $p$ $< 0.001$) and the DSS ($M=3.40$, $p$ $< 0.001$), $F(2,275)$ = 23.767, $p$ $< 0.001$, $\eta ^2_G=0.147$. Against the background that humans are always held legally liable for the final decision, and the fact that it is virtually impossible to assign legal responsibility to computer code, it seems plausible that more responsibility is attributed to humans. However, it is worth noting that AI is not given more responsibility than DSS—irrespective of AI’s self-learning nature.

When changing the perspective to being assessed, the result pattern also changed. Even though it is in line with our a priori assumptions, the fact that participants were more willing to be assessed exclusively by either an AI ($M=3.68$, $p$ $= 0.004$) or a DSS ($M=3.93$, $p$ $< 0.001$) compared to a human ($M=2.79$), $F(2,275)$ = 8.836, $p$ $< 0.001$, $\eta ^2_G=0.060$, contradicts the results mentioned above. Perhaps, a reason why the change of perspective went along with a change of preference was that when oneself is being assessed, one wants to be assessed as fairly and with as little personal bias as possible. A presumed major advantage of technical systems compared to humans is their objectivity and consistency^27–29^. This might have led participants to prefer the technical systems for their own assessment. As expected, participants were more willing to be assessed by a correct ($M=3.68$) compared to a faulty ($M=3.24$) system, $F(1,275)$ = 3.908, $p$ $= 0.049$, $\eta ^2_G=0.014$.

We contemplated whether the results (i.e., a general preference for humans over technical systems, except when being assessed oneself) might be related to the specific task context we used. In contrast to our experiment, previous research mostly used visual detection tasks, so perhaps the type of task (i.e., more verbalized, language-based task in Experiment 1) made a difference here. In order to clarify whether the results were due to the task context, we used an image classification task in Experiment 2. The personal context of personas was held constant to enable comparability between the experiments and only the type of task was changed.

### Experiment 2: X-Ray assessment

The task was changed to an X-Ray setting were participants had to estimate the amount of malignant cells in simulated X-Ray scans.

The X-Ray assessment task revealed the same pattern of results as the loan assessment task. First, and in line with the theoretical predictions, failure experience led to decreased agreement with the recommendations, $F(1,262)$ = 13.400, $p$ $< 0.001$, $\eta ^2_G=0.049$, perceived reliability, $F(1,262)$ = 5.888, $p$ $= 0.016$, $\eta ^2_G=0.022$, trust $F(1,262)$ = 6.621, $p$ $= 0.011$, $\eta ^2_G=0.025$, and willingness to be assessed, $F(1,262)$ = 6.939, $p$ $= 0.009$, $\eta ^2_G=0.026$. Again, participants interacting with different support agents did not differ in their forgiveness towards failures of the respective agent.

Second, the overall level of perceived reliability was again significantly ($t(267)=15.57, p<0.001$) lower (77.04%) than the true experienced reliability of the support. Keep in mind here that the true reliability was either 90% (failure condition) or even 100%. This is particularly surprising for the technical systems, as the task (i.e., image processing) was well-suited for automated analysis. This gap between perceived and actual reliability of technical systems might be a reason why human technology interaction cannot realize its symbiotic potential and partially explain that often times, human-technology joint performance is worse than what the technology can achieve by itself^30–33^. Third, despite the fit of task characteristics and technical agents, and against the predictions from the perfect automation schema, participants trusted the human ($M=82.60$) more than both the AI ($M=65.74$, $p$ $< 0.001$) and the DSS ($M=69.57$, $p$ $< 0.001$), $F(2,262)$ = 17.408, $p$ $< 0.001$, $\eta ^2_G=0.117$. Moreover, in line with the results of Experiment 1, more responsibility was attributed to the human ($M=5.07$) compared to the AI ($M=2.68$, $p$ $< 0.001$) and the DSS ($M=2.99$, $p$ $< 0.001$), $F(2,262)$ = 49.254, $p$ $< 0.001$, $\eta ^2_G=.273$.

Finally, the change of perspective led to a reversal of the support preference $F(2,262)$ = 4.911, $p$ $= 0.008$, $\eta ^2_G=0.036$. Participants were more willing to be assessed exclusively by a DSS ($M=3.10$) compared to a human ($M=2.19$, $p$ $= 0.009$). Descriptively, the willingness of exclusive evaluation by a DSS was higher than by an AI ($M=2.35$, $p$ $= 0.054$), whereas no difference was found between the AI and the human ($p$ $>0.999$). As in the case of loan assignment, technological judgment (i.e., DSS) was preferred over human judgment.

Again, despite using a task which resembled classical detection tasks, we did not find evidence for a preference for technical systems from the perspective of (artificial) colleagues. Perhaps, one possible reason for this might be that we again used personas with personal information shown along with the X-Ray images. Specifically, participants might have felt that support from another human is best-fitting in this personalized setting, despite the task characteristics itself (i.e., detection/scanning task). Thus, we conducted Experiment 3 in a task context which did not include any personal information at all.

### Experiment 3: chemical plant

In our third experiment, we used a simulated chemical plant task (i.e., estimation of container reactivity) to again study a visual detection task but without any personalized information.

Failure experience negatively influenced all relevant outcome variables (all *p* < 0.016). Overall perceived reliability was again quite low (74.64%) when considering the highly reliably support. It also significantly differed from the true reliability ($t(273)=15.96, p<0.001$). This underestimation—and a possibly resulting disuse—can have negative consequences in all presented domains including financial, medical, and public safety-critical decisions. Once more, participants trusted the human ($M=78.75$) more than the AI ($M=66.75$, $p$ $< 0.001$) as well as the DSS ($M=67.13$, $p$ $< 0.001$), whereas no trust difference between both technical systems was found ($p$ $>0.999$), $F(2,268)$ = 8.953, $p$ $< 0.001$, $\eta ^2_G=0.063$. Consistent with both other experiments, and quite contrary to our expectations, the present findings could well be coined as an *imperfect automation schema*. Furthermore, the human ($M=4.42$) was perceived as more responsible than the AI ($M=2.81$, $p$ $< 0.001$) and the DSS ($M=2.80$, $p$ $< 0.001$), $F(2,268)$ = 25.377, $p$ $< 0.001$, $\eta ^2_G=0.159$.

When asking the participants to change the perspective to being the one assessed, in this experiment, we asked participants to imagine living close to a permanent container storage which will open soon, as oneself being evaluated directly is not possible within this scenario. Regardless, the pattern of results was quite similar to that obtained in the previous two experiments. Here, participants were more willing to be judged exclusively by an AI ($M=3.30$) than by a human ($M=2.35$, $p$ $= 0.006$), $F(2,268)$ = 5.059, $p$ $= 0.007$, $\eta ^2_G=0.036$. The willingness to be judged by a DSS ($M=3.03$) did not differ from either other support agent (*p* > 0.108).

### Between-experiment comparison

Although a consistent pattern of results emerged throughout all three experiments, the level of ratings seem to differ between the different tasks. For this reason, we conducted a between-experiment comparison to investigate the still under-researched field of contextual influences (Experiment 1: complex categorization with personal information, Experiment 2: classical detection task with personal information, Experiment 3: classical detection task without personal information) and included the factor experiment as an additional factor in the ANOVA along with support agent and failure experience, resulting in a 3 (task context) x 3 (support agent) x 2 (failure experience) ANOVA. As the deviation of system recommendation is not comparable across contexts, we limited the analysis to the remaining key variables.

Participants perceived the support as more reliable in the loan assignment task ($M=84.66$) compared to both detection tasks—the X-Ray assessment ($M=77.04$, $p$ $< 0.001$) and chemical plant task ($M=74.64$, $p$ $< 0.001$), $F(2,805)$ = 27.976, $p$ $< 0.001$, $\eta ^2_G=0.065$. This is surprising as technical systems such as AI and DSS seem particularly well-suited for signal detection tasks (cancer detection, image analysis) rather than complex decision tasks with personal information (loan assignment)^3,22,23^. However, participants might not have considered the task-fit of technical systems but rather considered their own perceived task difficulty when judging perceived reliability.

Irrespective of task characteristics, the situational risk is likely a key contributing factor to these context differences. Specifically, an event that involves financial matters is perceived as less risky than an event involving health concerns^34^ or public safety concerns. Moreover, the failure effect was also less pronounced in the loan decision task for perceived reliability, $F(2,805)$ = 3.172, $p$ $= 0.042$, $\eta ^2_G=0.008$. This further supports the assumption that the expected consequences of failures had an influence.

Participants trusted the support more when working together on the loan assignment task ($M=79.72$) compared to both the X-Ray assessment ($M=72.67$, $p$ $< 0.001$) and the chemical plant task ($M=70.91$, $p$ $< 0.001$), $F(2,805)$ = 17.737, $p$ $< 0.001$, $\eta ^2_G=0.042$. This makes sense as there is usually a close link between trust and perceived reliability, however, it is still interesting to see that in the image processing tasks, regardless of personal information, trust towards any agent is generally lower. In addition and in line with reliability, the negative effect of failure experience was present in the X-Ray assessment ($p$ $= 0.008$) and chemical plant task ($p$ $< 0.001$), but not in the loan assignment task ($p$ $= 0.118$), $F(2,805)$ = 3.223, $p$ $= 0.040$, $\eta ^2_G=0.008$. The results of responsibility aligns well with the findings obtained for perceived reliability and trust. That is, participants ascribed more responsibility to the respective support in the loan assignment task ($M=4.03$) compared to the X-Ray assessment ($M=3.59$, $p$ $= 0.006$) and the chemical plant task ($M=3.35$, $p$ $< 0.001$), $F(2,805)$ = 11.636, $p$ $< 0.001$, $\eta ^2_G=0.028$.

Finally, the willingness to be assessed also changed with task context, $F(2,805)$ = 17.751, $p$ $< 0.001$, $\eta ^2_G=0.042$. In line with the other context effects, participants were more willing to be assessed themselves in the loan assignment task ($M=3.48$) than in the X-Ray assessment ($M=2.53$, $p$ $< 0.001$) and chemical plant task ($M=2.89$, $p$ $< 0.001$), no difference was found between the X-Ray assessment and chemical plant task ($p$ $= 0.112$). This might perhaps be related to the fact that even though a wrong evaluation in a loan decision context is highly displeasing, it is not as life-threatening as a wrong evaluation of an x-ray scan or reactive containers stored close to your home.

Taken together, a consistent picture emerged, showing that the support in the loan decision task was not only perceived as more reliable, trustworthy, and responsible, but also preferred for the judgment of oneself compared to both detection tasks. Notably, no differences occurred between the X-Ray and chemical plant task contexts which were similar in the task characteristics but dissimilar in their degree of personalization. Thus, the type of task (and potentially, its perceived risk) and not the degree of personalization seems to make a difference. Moreover, the results might also be associated with the perceived difficulty and comprehensibility of the task on the part of the participant. In comparison to the mathematically deterministic demands of the X-Ray and chemical plant task, the more natural language-based decision-making process in the loan assessment task might be viewed as easier for the participants^23,35^. Despite the consistency of the findings, they still have to be taken with a grain of salt, as the interaction with the respective agent was limited to a rather short online experiment. Moreover, as the a priori sample size was determined on a single-experiment basis, the between-experiment comparison of the different contexts was likely over-powered. This is particularly relevant for the interaction effects, which were rather small, and therefore should be interpreted with caution. However, for the main effects, given that the effect sizes were mostly medium, the majority of the main effects of context would have also been obtainable with a smaller sample size. Regardless, the present results can still be an interesting starting point for future research and illustrate the need to consider multiple contexts and perspectives. Specifically, future research needs to corroborate the present results with longer human-agent interaction and ideally even real systems, given that the interaction in our experiments was quite short.

## Discussion

In direct contrast to the widely held assumption of a perfect automation schema in human-automation interaction, the findings hint in the opposite direction, i.e., to an *imperfect automation schema*. Moreover, the results suggest that this holds true for both classical automation and novel technological advancements like AI. It seems peculiar that there were virtually no differences between the two types of technological systems. One might wonder whether both systems are generally just not perceived differently from the general public. Specifically, both types of systems share some key commonalities which might be much more salient than how a technical system makes its decision—likely, both AI and DSS might be viewed as an opaque black box. Nonetheless, this assumption needs to be further corroborated in future online and offline research. If future research further confirms the present indifference between the perception of AI and DSS, then anyone working on future implementations of AI into virtually any field can learn from the many earlier findings^18,36^ and theoretical models^8,9,37^ of human-automation interaction.

The most puzzling finding obtained consistently through three experiments was that when asking participants to change the perspective from an advice-taker to the person being assessed, the preference for humans over technical systems reversed. Perhaps, when working together, factors like a broad subject matter expertise and intuitiveness might be more relevant^10^. In contrast, when being assessed, these factors might not be as relevant—tools tailored to a specific sub-task, their case-by-case performance, and lack of personal biases might determine the willingness to be assessed by machine intelligence. Specifically, a presumed major advantage of technical systems compared to humans is their objectivity and consistency^27–29^. A common fear when being judged by a technical system is reductionism of the algorithm, i.e., that unique individual factors might not be considered^38,39^. However, this reductionism is also what makes technical systems less biased than humans^29^ and might also be perceived as a strength when being assessed oneself. This finding should serve as food for thought, as new technological advancements require both more and more workers to collaborate with artificial colleagues as well as more and more persons being exposed to artificial assessors.

## Method

All experiments were preregistered via the OSF. Experiments were approved by the ethics committee at the Department of Psychology and Ergonomics, Technische Universität Berlin. All research was performed in accordance with relevant guidelines/regulations and with the Declaration of Helsinki. Participants gave their informed consent prior to participation.

### Participants

The experimental data was collected sequentially and participants were only allowed to participate in one of the experiments. All participants were recruited within a six-month time frame between November 2020 and April 2021. The same criteria of participant selection were used for all three experiments. This procedure and the close spacing of data collection in time should ensure that the samples recruited for each data collection were drawn from the same population. Altogether, we recruited 352 participants in Experiment 1, 348 in Experiment 2, and 334 in Experiment 3. After applying an attention check, this resulted in 300 participants in each experiment, who were randomly assigned to one of the six experimental conditions in equal numbers. A sample size of 300 participants per experiment was targeted to obtain close to .90 power to detect a small to medium effect size of .20 at the standard .05 alpha error probability. Moreover, we also performed a manipulation check in the failure condition (i.e., excluding all participants who exactly followed the obviously incorrect advice) which led to an additional exclusion of 19 participants in Experiment 1, 32 participants in Experiment 2, 26 participants in Experiment 3. This additional check was done because it is impossible to separate a lack of attention from overtrust in this experiment. Each experiment took about 10 minutes in total and participants were reimbursed 1.40£.

In Experiment 1, the final sample consisted of 281 (mean age = 33.48, *SD* = 5.59, 42% female) participants. The participants in each condition did not differ in their disposition to trust technology (*p* = 0.989). In Experiment 2, the sample consisted of 268 participants (mean age = 33.35, *SD* = 6.24, 49% female) and there were no differences for the control variable disposition to trust technology between experimental groups (*p* = 0.662). In Experiment 3, the final sample contained 274 participants (mean age = 33.33, *SD* = 4.94, 35% female) with again no differences in the control variable disposition to trust technology between the experimental conditions (*p* = 0.252).

### Procedure

All experiments were programmed in jspsych^40^ and run on a jatos server^41^. In all three experiments, participants gave their informed consent and were briefed about the general aim of the study. Subsequently, the experiments started with a general introduction to the task. Then, participants were shown three written examples of previous scenarios, together with the decision made at that time including feedback about how the decisions later turned out (decisions were always correct in the examples). The interface used to display the examples was the same as used later in the experiment when the participants had to make their own decisions in interaction with the support agent (despite the feedback on the correctness).

After these example scenarios, participants were informed that for their own decisions, they would be supported by either an AI, a DSS, or an experienced colleague. They were told that the respective agent took in all the information provided in the examples they saw earlier along with some additional information. In addition, some further information about their support agent was provided. Specifically, in the AI condition, participants were informed that the AI was an innovative technology which made its decisions based on deep neural networks and learned parameters. In the DSS condition, the DSS was framed as a well-established decision support system based on prior loan data which made its decisions based on predefined parameters and a fixed algorithm. In the human condition, the colleague was framed as a colleague with considerable experience with many previous cases and knowledgeable to decide which parameters one needs to pay attention to. Independent of the kind of support, all participant were informed that their support agent was more than 90% reliable and that decisions in the respective scenario were nowadays widely made with support of the respective support agent. After this framing information, two attention check questions were asked. They included two questions in order to check whether participants had correctly understood the specific definition of their condition. The first one was related to what type of support agent were giving the recommendations to the participant. The second one related to the characteristic of the respective agent.

**Figure 2:**
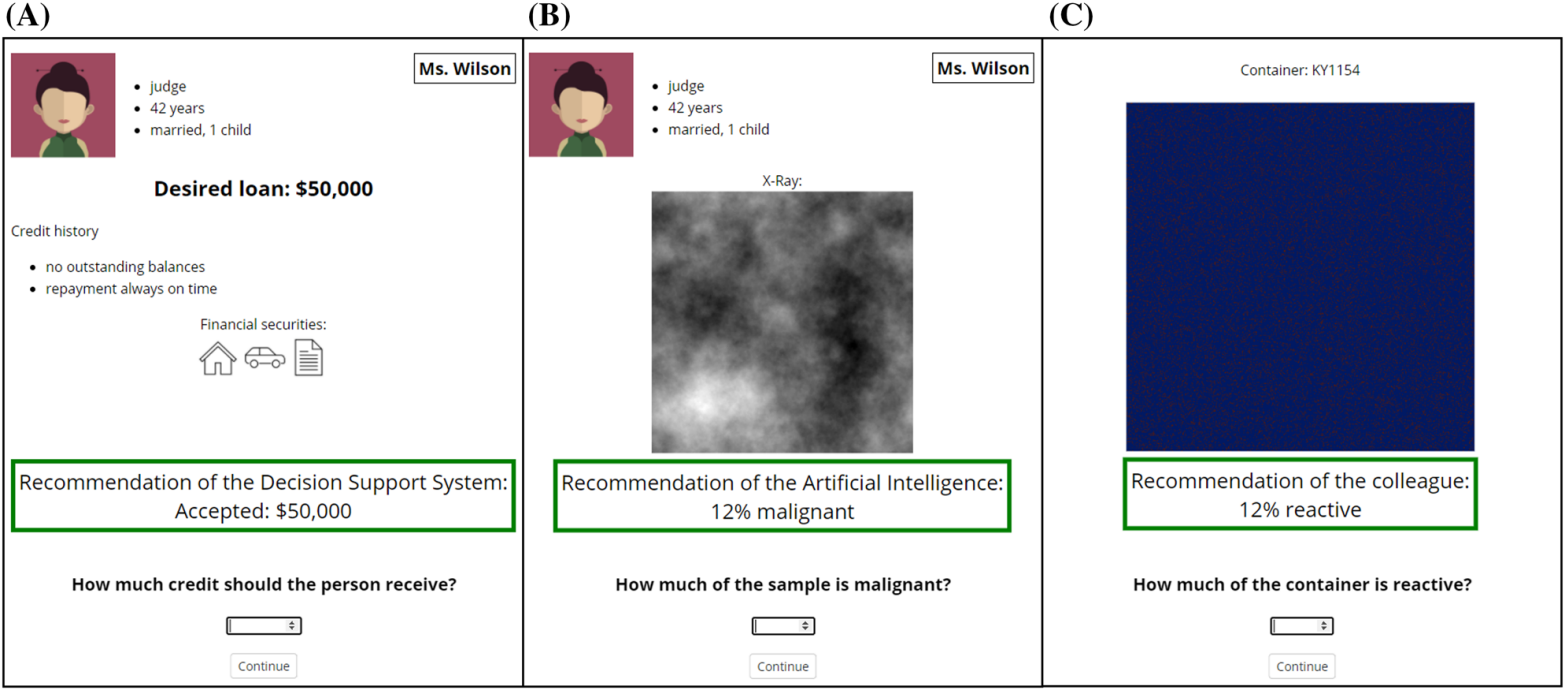
Depiction of the second trial in the decision support system condition in the context loan assignment of Experiment 1 (**A**), the artificial intelligence condition in the context radiology of Experiment 2 (**B**), and the human condition in the chemical plant task context of Experiment 3 (**C**).

Subsequent to the attention check, participants received information on how the task would look like and were familiarized with the interface and its function. Then participants made their own decisions with support of their respective agent in ten trials of the respective task. In all experiments, the trial structure was as follows: for 5 seconds, participants were shown all information except the agent’s recommendation with the note that the support agent (i.e., AI, DSS, or human) were processing (AI and DSS) or evaluating (human) the information. After 5 seconds, participants were told to press the space-bar to continue if they were ready to see the recommendation of the agent. Upon key press, the recommendation was shown with an input field at the bottom of the page for their final decision. Examples of the interfaces displaying the recommendation in the different experiments are shown in Fig. 2. After each trial, participants were asked to press the space-bar to continue to the next trial. In the failure condition, all recommendations except one recommendation were correct. That is, in trial 7, the support agent gave an obviously wrong recommendation. In the correct condition, all recommendations were perfectly reliable. After all 10 trials, participants were asked to fill out a short questionnaire about their interaction experience.

The experiments differed with respect to the specific information provided as basis for the decision-making in the different scenarios, as well as the kind of recommendation provided by the support agent. Specifically, in Experiment 1, the task was to assess the creditworthiness of personas. The experiment started with a general introduction on loan assignment and what typical factors are considered by banks before making a loan decision (e.g., income, debts, etc.). Then participants worked on ten trials where fictional personas applied for loans. Personas differed in their name, age, job, family status, outstanding debts, securities, and desired loan. Recommendations from the respective agent for loan grants were color coded (i.e., green: fully, orange: partially, red: rejected). In trial 7 of the failure condition, the support agent recommended granting a full loan to a persona without a job and with lots of debt.

In Experiment 2, the task was to evaluate which percentage of simulated X-Rays were brighter than a given cutoff (brighter than gray-scale value of 150). We used simulated $1/f^3$ noise as stimulus material as this resembles the power spectrum of real-world mammograms^42^. To enable participants performing this task, the experiment started with an introduction where they were first shown a gray-scale continuum, with the critical cutoff threshold marked, along with an example image. Participants were instructed that parts brighter than this could possibly be malignant and that later on, they would be asked to estimate the percentage of potentially malignant tissue in X-Ray samples. They were also instructed that this cutoff was very cautious, and that there is typically no reason for concern at all if the percentage is lower than 15%. The hypothetical X-Ray scans were embedded in the same personas as in Experiment 1 in regard to their name, age, job, and family status. Again, recommendations were color coded (i.e., green: < 15% malignant, orange: > 15% < 50% malignant, red: > 50% malignant). In trial 7 of the failure condition, a bright image (59%) was evaluated to be okay (8% malignant).

The task in Experiment 3 was to estimate the percentage of potentially reactive particles of the fictional mineral *Rorigium* at a simulated chemical plant. Specifically, the task was to evaluate the percentage of orange pixels in images which had orange and blue pixels with a fixed proportion of orange pixels which varied from trial to trial. At the beginning of the experiment, participants were told that each image represented one chemical container of the fictional element *Rorigium* which was considered to be moved to a permanent storage (or needed further treatment prior to that). Participants were told that the higher the percentage of orange pixels, the more reactive the respective container. To keep Experiment 3 as parallel as possible to Experiment 2, participants were again told that if the percentage is below 15%, the containers can be safely stored. All percentages of orange pixels were the same as the percentages of pixels brighter than the cutoff in Experiment 2. In the examples and in the task itself, the personas used in the first two experiments were replaced with neutral information in order to remove any direct personal involvement from the decision-task (i.e., only a serial number of the container was shown above each image). Recommendations were again color-coded (i.e., green: < 15% reactive, yellow: > 15% < 50% reactive, red: > 50% reactive). In trial 7 of the failure condition, an orange-dominant image (59% orange pixels) was evaluated to be okay (8% orange pixels).

### Design and dependent variables

Each experiment consisted of a 2 (failure experience: one vs. none) × 3 (interaction agent: AI vs. DSS vs. human) between-subjects design. The dependent measures were kept the same for all experiments. As an objective indicator of compliance with the recommendation, we measured the participants’ *deviation of the recommendation* by the respective agent during the ten trials. In the subsequent questionnaire we then asked participants to indicate how reliable they perceived their support agent to be (0-100%) to measure *perceived reliability*. *Trust* was also assessed via a single item: participants were asked how much they trusted their support agent (0 (*not at all*) to 100 (*completely*)).

As one reviewer noted, the validity of a single-item trust measure was unclear in an earlier version of this manuscript compared to better validated and widely used trust questionnaires. To address this, we replicated the agent-effects in the all-correct condition of Experiment 2 using both our single-item trust as well as the questionnaire by Jian and colleagues^43^ and counterbalanced the order of the single-item and the questionnaire. We obtained the same results with both approaches (i.e., main effect of support agent with significant differences in follow-up Bonferroni-corrected pairwise tests for the human vs. DSS ($ps<0.019$) and the human vs. AI ($ps<0.006$) comparisons but not in the AI vs. DSS comparisons) and a high correlation between the two trust measures ($r=0.841, p<0.001$). We are therefore confident that the single-item can capture similar effects on trust as one of the most widely used questionnaires in the field. Besides these global subjective judgments on reliability and trust, participants also rated the perceived involvement of the support agent in the decision via an adopted version of the causal attribution scale^44^. Here, the last item was used to measure *responsibility* of the respective agent. More precisely, participants were asked to rate if the respective agent took responsibility for its actions on a seven-point Likert scale from *strongly disagree* to *strongly agree*. In addition, the participants were asked to change their perspective from collaboration to being assessed oneself. Specifically, they were asked whether they themselves would like to be judged exclusively by such type of system. This was assessed on a seven-point Likert scale from *definitely not* to *definitely* to measure *the willingness to be assessed oneself*. Note that this question involved the change of perspective from being a user of the respective support agent to becoming dependent on it as the person affected by its decision.

To prevent confounding effects of participants’ attitudes towards technology the disposition to trust technology^45^ was measured as a control variable at the end of the experiment. The questionnaire^45^ consisted of three items considering the general trust towards new information technologies (e.g., “I usually trust in information technology until it gives me a reason not to.”), which were rated on a seven-point Likert scale from *strongly disagree* to *strongly agree*.

Some additional outcomes were measured but were not reported here for conciseness (please refer to the OSF for the full data). These outcomes included two questions on perceived usefulness and the first three items of the causal attribution scale^44^.

### Statistics

The data of the three experiments was analyzed in two stages. First, analyses were conducted on the level of the individual experiments. Therefore, the control variable disposition to trust technology was compared between all six experimental conditions for each experiment via a one-way between-subjects ANOVA to check for intergroup differences. Furthermore, for all outcome variables of the respective experiment two-way between-subjects ANOVAs were conducted to investigate main effects of failure experience (i.e., failure experience vs. all-correct suggestions) and support agent (i.e., AI vs. DSS vs. human), as well as the interaction effect of both factors. Second, the between-experiments comparison was performed via three-way (i.e., context, support agent, failure experience) between-subjects ANOVAs to examine the main effect of the interaction context and possible interactions of this factor with failure experience and support agent. For all analyses, conventional values including F statistic, p-values and generalized eta-square are reported. In addition, for all intra- and inter-experimental analyses in which either a significant interaction effect or main effect of a three-level factor occurred, Bonferroni-corrected post-hoc test were conducted.

## Data Availability

Raw data, analysis scripts, and experiment files can be obtained via the Open Science Framework at https://osf.io/c5tsj/. All experiments were preregistered (Experiment 1: https://osf.io/kbfvn, Experiment 2: https://osf.io/eyf8m, Experiment 3: https://osf.io/pxq4k, comparison between experiments: https://osf.io/y7fpw).
